# Effects of cyclosporine on ischemia-reperfusion injuries in rat kidneys. An experimental model ^1^

**DOI:** 10.1590/s0102-865020190080000006

**Published:** 2019-10-14

**Authors:** Antonio Carlos Cerqueira Oliveira, Norma Sueli Pinheiro Módolo, Maria Aparecida Custódio Domingues, Paulo Adriano Schwingel

**Affiliations:** I MSc, Department of Anesthesiology and Surgery , Complexo Hospitalar Universitário Professor Edgard Santos (HUPES), Universidade Federal da Bahia (UFBA), Salvador - BA , Brazil . Conception and design of the study; acquisition, analysis and interpretation of data; technical procedures; manuscript writing; final approval.; II PhD, Full Professor, Department of Anesthesiology , Faculdade de Medicina de Botucatu (FMB), Universidade Estadual Paulista Júlio de Mesquita Filho (UNESP), Botucatu - SP , Brazil . Conception and design of the stusy, analysis and interpretation of data, manuscript preparation, critical revision, final approval.; III PhD, Assistant Professor, Department of Pathology , FMB , UNESP , Botucatu - SP , Brazil . Conception and design of the study, analysis and interpretation of data, manuscript preparation and writing.; IV PhD, Associate Professor, Human Performance Research Laboratory (LAPEDH), Universidade de Peranambuco (UPE), Petrolina - PE , Brazil . Analysis and interpretation of data, manuscript writing, critical revision, final approval.

**Keywords:** Acute Kidney Injury, Cyclosporine, Kidney, Rats, Wistar

## Abstract

**Purpose:**

To assess Cyclosporine A (CsA) therapy at an intraperitoneal dose of 15 mg.kg ^-1^ in a rodent model of non-septic renal ischemia.

**Methods:**

Twenty male Wistar rats were randomized to receive CsA therapy or none therapy before undergoing 30 minutes of renal ischemia followed by reperfusion. Additionally, 10 rats were randomized to undergo the same surgical procedure of the aforementioned animals with neither ischemia nor CsA therapy. Twelve hours after kidney ischemia, the left kidneys were evaluated for histological injury according to Park’s criteria. Serum creatinine (Cr), urea nitrogen (Ur) and sodium levels were obtained at different times of the experimental protocol.

**Results:**

Rodents in the CsA group showed negative results (p<0.05) in serum variables (Cr: 0.41±0.05mg/dL *vs* . 4.17±1.25mg/dL; Ur: 40.90±3.98mg/dL *vs* . 187.70±22.93mg/dL) even the non CsA or control group (Cr: 0.35±0.07mg/dL *vs* . 3.80±1.20mg/dL; Ur: 40.10±4.70mg/dL *vs* . 184.50±49.80mg/dL). The negative results were also verified in histological evaluation, CsA group had 50% in the very severe grade of lesion, 10% in the severe and 40% in the moderate to severe whereas the control group had 90% in the very severe grade.

**Conclusion:**

CsA was incapable of preventing the deleterious effects of ischemia-reperfusion injury in rat kidneys.

## Introduction

Ischemic injuries in vital organs, such as the heart, brain and kidneys, can decisively contribute to increased morbidity and mortality ^1 , 2^ . Renal ischemia during arterial occlusion, shock and organ transplantation are commonly associated with cell death, renal failure, delayed graft function in kidney transplantation and renal graft rejection ^3^ . Following an episode of acute renal ischemia, early reperfusion continues to be a first-line strategy to limit damage caused to this organ. Nonetheless, renal reperfusion *per se* has the potential to cause cellular death ^4^ , similarly to what has been observed in the heart ^5^ . An investigation of protective strategies utilized at the time of reperfusion is fundamental to preventing this type of lesion ^6 , 7^ .

Epidemiological data on acute kidney injury (AKI) have proven to be controversial for some time, i.e. an enormous variation exists in the rate of incidence, which ranges between 1 and 31%, while mortality rates can vary as much as 28 to 82%. This has been primarily due to assorted ways of defining AKI – largely resolved following the publication of standardized diagnostic RIFLE (Risk, Injury, Failure, Loss of kidney function and End-stage kidney disease) criteria in 2004 ^2^ . Regardless, methodologic variability in articles attempting to assess the effect of cyclosporine A (CsA) on renal injury remains considerable ^8 , 9^ .

Several intervention strategies to treat ischemia-reperfusion injury (IRI) have been proposed, one of which is the use of CsA ^10 , 11^ . CsA, an immunosuppressive drug routinely employed in solid organ transplantation, offers a protective effect attributed for some organs. Cardiac-associated CsA use has been investigated since the 1990s, and research focused on its ability to reduce the extent of myocardial infarction following myocardial ischemia-reperfusion has reported satisfactory outcomes ^12^ . On the other hand, the literature is controversial with respect to IRI in the kidney largely due to a dearth of investigation ^10 , 13^ .

Given that CsA demonstrates potential to protect against acute ischemic injury in some organs and considering that there is an absence of consensus with respect to its protective role in the kidney, the present study aimed to evaluate the effects of CsA on renal IRI in rats.

## Methods

The present experimental study was conducted between March and June 2013. Our research proposal received approval from the Animal Experimentation Institutional Review Board (number 1034-2013, later modified by CEUA number 24/2015) to include 30 male Wistar rats weighing no less than 300 grams each, provided by the Central Animal Care Facility. These animals were randomly divided by using a computer-generated table of random number into three groups: Sham Group (SG), submitted to laparotomy and right nephrectomy; Control Group (CG), also submitted to laparotomy and right nephrectomy, in addition to ischemia-reperfusion of the left kidney; and CsA Group (CsAG), submitted to the same procedures as the above groups, and that additionally received intraperitoneal (IP) CsA at a dose of 15 mg.kg ^-1^ , in two different moments, 24 hours before and immediately preceding the first surgical intervention.

### Outcomes

Systolic (SBP), diastolic (DBP) and mean (MBP) blood pressures, as well as rectal temperature (T), were evaluated as measures of organic homeostasis maintenance (physiological variables). In addition, serum levels of sodium (Na), urea (Ur) and creatinine (Cr) were measured (serum variables). A histological examination of the kidneys was conducted according to Park’s criteria ^14^ ( Table 1 ). Creatinine measures were used in the RIFLE criteria for AKI ( Table 2 ).

**Table 1:** Histological slide demonstrating renal tubular necrosis marks in this present study which supports the Park Evaluation (image is increased by x400). - Histopathology grading system used in kidney examination.

Grade	Type	Tubular necrosis (%)
0	No lesion	0
1	Mild	<10
2	Moderate	10 to 25
3	Moderate to severe	26 to 50
4	Severe	51 to 75
5	Very severe	>75

**Table 2:** RIFLE criteria for acute kidney injury.

	GRF	URINE OUTPUT (U.O.)	
RISK	Increased SCr X 1.5 or GFR decrease > 25%	U.O. < 0.5 mL/Kg/h x 6h	High sensitivity
INJURY	Increased SCr X 2 or GFR decrease > 50%	U.O. < 0.5 mL/Kg/h x 12h	
FAILURE	Increased SCr X 3 or GFR decrease > 75% or SCr ≥ 4.0 mg/dL or acute rise SCr ≥ 0.5 mg/dL	U.O. < 0.5 mL/Kg/h x 24h or anuria x 12h	
LOSS	Persistent acute renal failure = complete loss of kidney function > 4 weeks		High specificity
ESKD	End-stage kidney disease > 3 months		

Variables were measured at three time points: physiological at T0 – following catheterization of the left carotid artery; T1 – after clamping of the left renal artery; T2 – 30 minutes after releasing the left renal artery. The serum variables were measured at M0 = T0, M1 = T2, and M2 = 12 hours following the conclusion of the initial surgical procedure (Table 3).

Variables were measured at three time points: physiological at T0 – following catheterization of the left carotid artery; T1 – after clamping of the left renal artery; T2 – 30 minutes after releasing the left renal artery. The serum variables were measured at M0 = T0, M1 = T2, and M2 = 12 hours following the conclusion of the initial surgical procedure ( Table 3 ).

**Table 3:** Data collection moments during the evaluation process.

Carotid Dissection	Ischemia-Reperfusion Maneuver	Abdominal Reopening
T0	T1/T2	
M0	M1	M2

### Experimental sequence

All animals obtained from the Central Animal Care Facility were kept in an environment with controlled temperature, humidity, noise and sleep-wake cycle in metabolic cages with access to food and water *ad libitum* for 24 hours before surgery. At this time point, rats in the CsA group received the drug by IP route at a dose of 15.0 mg.kg ^-1^ . CsA was administered in a 2% sterile solution (Citopharma Manipulações Parenterais, Belo Horizonte, Minas Gerais, Brazil) and diluted in distilled water at a final volume of 1.0 mL.

After the initial 24 hours, each animal was weighed and anesthetized inside a bell jar containing an environment of 40% oxygen and 3 to 4% isoflurane. Following the induction of anesthesia, the animals were maintained under spontaneous ventilation via a mask adjusted to each animal’s mouth and snout, which was adapted to an avalvular device to effectively administer the anesthetic at a concentration of 1.5 to 3% with no carbon dioxide (CO _2_ ) gas absorber. After falling asleep and being completely anesthetized, the animals were placed on heated bags over a platform to maintain a rectal temperature between 37 and 39ºC while being monitored for temperature and systolic, diastolic and mean blood pressures via sensors connected to an AS/3™ Compact Anesthesia Monitor (Datex-Ohmeda Inc., Helsinki, Finland). At this time, all CsA group animals received a second dose of the drug (15 mg.kg ^-1^ via IP).

A cervical incision was made to dissect the right internal jugular vein in order to infuse saline (Ringer’s lactate) using an ANNE™ infusion pump (Abbott Laboratories, Lake Buff, IL, USA), in addition to another incision in the left carotid artery to allow cardiovascular monitoring and blood sample collection. Both vessels were catheterized using a 24-gauge Teflon cannula. Saline was infused immediately following venous dissection at a rate of 3 mL.h ^-1^ . Blood was then collected (1.0 mL) to measure creatinine, urea and sodium and a 3.0 mL bolus of saline was administered to maintain hemodynamic stability.

Laparotomy was performed via a vertical midline incision. Nephrectomy procedures were carried out following identification of the vascular pedicle of the right kidney. After identifying and dissecting the left renal artery, an atraumatic bulldog clamp was placed for 30 minutes. Thereafter, clamping was discontinued and 1.0 mL of blood was collected from the left carotid artery at this moment, followed by the infusion of a 3.0 mL saline bolus. The abdominal wall was then closed and surgical wound edges were treated with 0.125% bupivacaine without a vasoconstrictor as a postoperative analgesic. Arterial and venous catheters were removed and cervical incisions were closed. The anesthetic administration of isoflurane was then interrupted and each animal was clinically monitored until recovery.

Upon recovery all animals were returned to their metabolic cage environment for a period of 12 hours under conditions identical to those at the outset of experimentation. Right after, all animals were anesthetized using the same procedure described above, after which 1.5 mL of intracardiac blood was collected for Na, Ur and Cr quantitation. Animals were subsequently sacrificed by intracardiac administration of 0.5% bupivacaine without a vasoconstrictor at a dose of 3.0 mg.kg ^-1^ .

### Statistical analysis

The sample size calculation was made using the WinPepi (PEPI-for-Windows) version 11.63 ^15^ . Descriptive analyses were performed with respect to the following variables: systolic, diastolic and mean arterial pressure, sodium, urea and creatinine levels, weight, temperature, histology and RIFLE status. With respect to quantitative variables, homoscedasticity analysis was performed by histogram assessment, mean and median value comparison, Skewness and Kurtosis values, mean and standard deviation comparison and hypothesis testing, i.e. Kolgomorov-Smirnov. Inferential statistics were analyzed using one-way analysis of variance (ANOVA) with Bonferroni post-hoc testing to compare weight, SBP, DBP, MBP, T, creatinine, urea and sodium values between groups. With the exception of weight, repeated measures (RM) ANOVA was used to conduct paired analysis of measured variables among the three evaluation moments. A p-value ≤0.05 was used to reject the null hypothesis.

## Results

The statistical analysis demonstrated that the mean weight of Wistar rats from CG (458.0±50.5) was higher (p=0.002) than from CsAG animals (405.0±46.7), and then from SG rodents (387.0±18.9). In addition, rats from CsAG and SG have similar mean weights according to the Bonferroni’s post-hoc comparisons tests. One-way ANOVA revealed that the rectal T of the animals and the SBP values were similar among all three groups (p>0.05). On the other hand, MBP and DBP demonstrated statistical differences between the groups according to post-hoc tests ( Table 4 ).

**Table 4:** Comparison between groups of physiological features from Wistar rats (n=30) submitted to laparotomy and right nephrectomy.

Groups	T0	T1	T2
	(mean±SD)	(mean±SD)	(mean±SD)
***Mean Blood Pressure (mmHg)***			
SG (n=10)	98.50±13.69 ^a^	75.00±10.80 ^b^	83.00±12.29 ^b^
CG (n=10)	112.00±9.47 ^b^	72.00±12.99 ^a,b^	77.00±11.45 ^a,b^
CsAG (n=10)	96.00±9.51 ^a^	71.00±10.04 ^a^	66.00±15.60 ^a^
*p-value*	*0.007*	*0.035*	*0.020*
***Systolic Blood Pressure (mmHg)***			
SG (n=10)	119.00±16.14	95.00±8.23	103.00±9.53
CG (n=10)	133.00±10.44	96.00±13.08	104.00±15.26
CsAG (n=10)	122.00±10.64	86.00±8.37	90.00±11.36
*p-value*	*0.061*	*0.082*	*0.390*
***Diastolic Blood Pressure (mmHg)***			
SG (n=10)	88.00±13.24 ^b^	64.00±12.91 ^b^	83.00±9.25 ^b^
CG (n=10)	102.00±9.15 ^c^	60.00±13.81 ^a^	49.00±12.30 ^a^
CsAG (n=10)	73.00±14.12 ^a^	64.00±11.53 ^b^	53.00±18.43 ^a^
*p-value*	*0.001*	*0.040*	*0.023*
***Rectal Temperature (°C)***			
SG (n=10)	36.98±0.60	37.60±0.50	37.54±0.81
CG (n=10)	37.14±0.50	37.98±0.80	37.71±0.60
CsAG (n=10)	37.43±0.70	38.22±0.40	37.67±0.60
*p-value*	*0.347*	*0.205*	*0.837*

Different letters represent statistical differences between groups of Bonferroni post-hoc test.

SG: Sham Group; CG: Control Group (also submitted to ischemia-reperfusion of the left kidney); CsAG: Cyclosporine A Group (submitted to the same CG procedures plus intraperitoneal CsA at a dose of 15 mg.kg ^-1^ ); SD: standard deviation; T0: following catheterization of the left carotid artery; T1: after clamping of the left renal artery; T2: 30 minutes after releasing the left renal artery.

**Figure 1:**
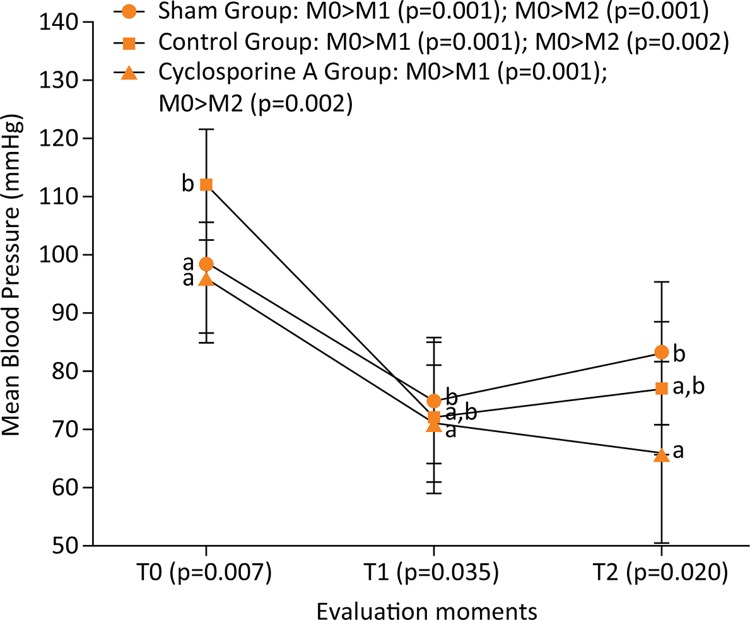
Comparison (intragroup analysis) between the three evaluation moments of mean blood pressure from Wistar rats (n=30) submitted to laparotomy and right nephrectomy.

Legend: Different letters represent statistical differences between groups (intergroup analysis) of Bonferroni post-hoc test. Control Group is also submitted to ischemia-reperfusion of the left kidney; Cyclosporine A Group is submitted to the same procedures of control group plus intraperitoneal Cyclosporine A at a dose of 15 mg.kg^-1^; T0: following catheterization of the left carotid artery; T1: after clamping of the left renal artery; T2: 30 minutes after releasing the left renal artery.

RM ANOVA revealed that there is a statistically significant difference between the three evaluation moments in the intragroup analysis. The ANOVA measurements in MBP (F(2.81)=64.52 and p<0.001) followed by Bonferroni’s post-hoc indicated that means in the M0 was higher than M1 for all groups. In addition, intragroup analysis revealed that the mean values of MBP in M0 was also higher than values observed in M2 for all groups ( Fig. 1 ).

**Figure 2:**
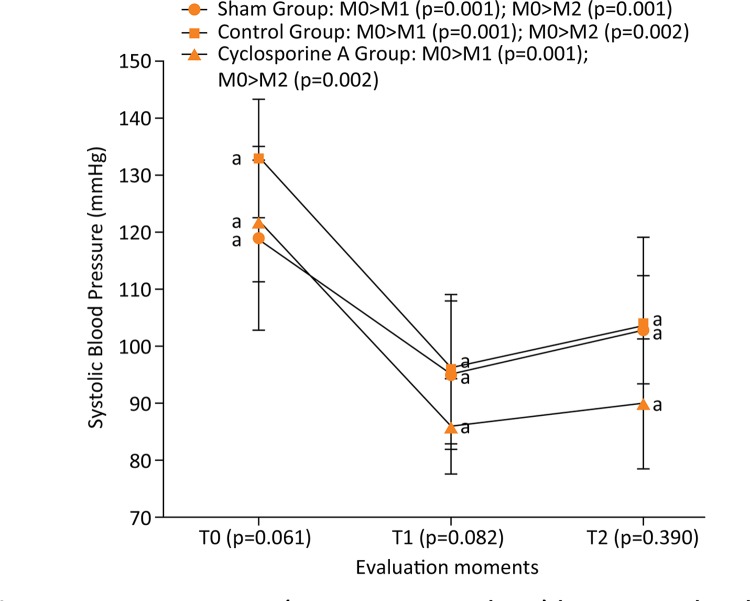
Comparison (intragroup analysis) between the three evaluation moments of systolic blood pressure from Wistar rats (n=30) submitted to laparotomy and right nephrectomy.

Legend: Different letters represent statistical differences between groups (intergroup analysis) of Bonferroni post-hoc test. Control Group is also submitted to ischemia-reperfusion of the left kidney; Cyclosporine A Group is submitted to the same procedures of control group plus intraperitoneal Cyclosporine A at a dose of 15 mg.kg^-1^; T0: following catheterization of the left carotid artery; T1: after clamping of the left renal artery; T2: 30 minutes after releasing the left renal artery.

In the same way, RM ANOVA measurements in SBP (F(2,81)=58.39 and p<0.001) followed by Bonferroni’s post-hoc indicated the same results. Means values from M0 were higher than M1 and M2 for all groups ( Fig. 2 ).

**Figure 3:**
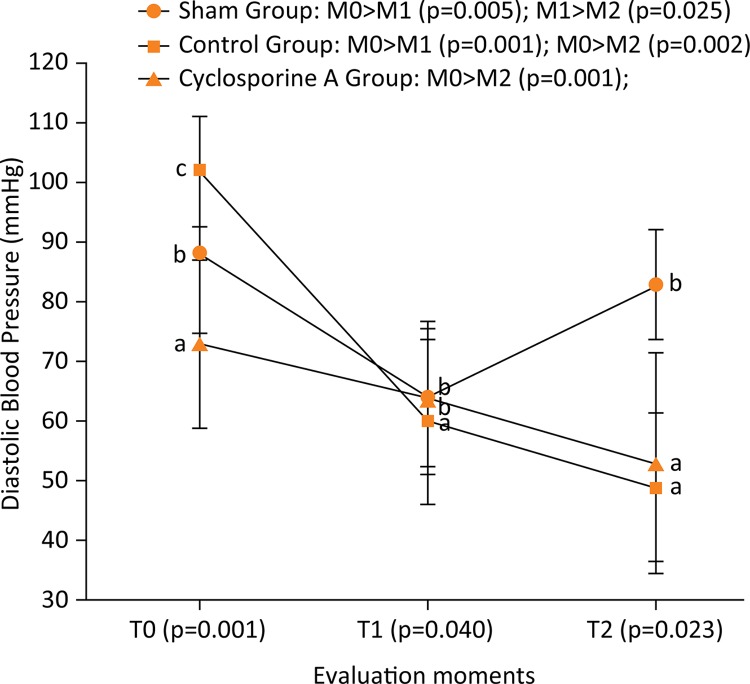
Comparison (intragroup analysis) between the three evaluation moments of diastolic blood pressure from Wistar rats (n=30) submitted to laparotomy and right nephrectomy.

Legend: Different letters represent statistical differences between groups (intergroup analysis) of Bonferroni post-hoc test. Control Group is also submitted to ischemia-reperfusion of the left kidney; Cyclosporine A Group is submitted to the same procedures of control group plus intraperitoneal Cyclosporine A at a dose of 15 mg.kg^-1^; T0: following catheterization of the left carotid artery; T1: after clamping of the left renal artery; T2: 30 minutes after releasing the left renal artery.

Results from RM ANOVA for DBP (F(2.81)=38.41 and p<0.001) followed by post-hoc tests indicated that the values from M0 were higher than M1 in SG and CG. Means values from M0 were also higher than from M2 in CG and CsAG using intragroup analysis. All results for DBP over the three time points evaluated are presented in the Figure 3 .

With respect to sodium levels, no significant differences were seen among groups in the intergroup analysis for the three time points evaluated ( Table 5 ): SG (p=0.291), CG (p=0.064), CsAG (p=0.367).

**Table 5:** Comparison between groups of serum variables from Wistar rats (n=30) submitted to laparotomy and right nephrectomy.

Groups	M0 (or T0)	M1 (or T2)	M2
	(mean±SD)	(mean±SD)	(mean±SD)
***Creatinine (mg/dL)***			
SG (n=10)	0.35±0.05 ^a^	0.47±0.06 ^a^	0.62±0.06 ^a^
CG (n=10)	0.35±0.07 ^a^	0.80±0.18 ^b^	3.80±1.20 ^b^
CsAG (n=10)	0.41±0.05 ^a^	0.87±0.08 ^b^	4.17±1.25 ^b^
*p-value*	*0.060*	*<0.001*	*<0.001*
***Urea (mg/dL)***			
SG (n=10)	44.40±4.52	47.40±8.23	43.60±7.39 ^a^
CG (n=10)	40.10±4.70	48.40±5.30	184.50±49.80 ^b^
CsAG (n=10)	40.90±3.98	52.20±4.89	187.70±22.93 ^b^
*p-value*	*0.890*	*0.900*	*<0.001*
***Sodium (mg/dL)***			
SG (n=10)	36.98±0.60	37.60±0.50	37.54±0.81
CG (n=10)	37.14±0.50	37.98±0.80	37.71±0.60
CsAG (n=10)	37.43±0.70	38.22±0.40	37.67±0.60
*p-value*	*0.911*	*0.124*	*0.753*

Different letters represent statistical differences between groups of Bonferroni post-hoc test.

SG: Sham Group; CG: Control Group (also submitted to ischemia-reperfusion of the left kidney); CsAG: Cyclosporine A Group (submitted to the same CG procedures plus intraperitoneal CsA at a dose of 15 mg.kg ^-1^ ); SD: standard deviation; M0: following catheterization of the left carotid artery; M1: 30 minutes after releasing the left renal artery; M2: 12 hours following the conclusion of the initial surgical procedure.

RIFLE criteria evaluation indicated negative results for CG and CsAG compared to SG ( Table 6 ).

**Table 6:** Descriptive analysis of RIFLE criteria among Wistar rats (n=30) submitted to laparotomy and right nephrectomy.

RIFLE criteria	GROUPS		
	SG (n=10)	CG (n=10)	CsAG (n=10)
No risk, n (%)	-	1 (10.0%)	-
Risk, n (%)	6 (60.0%)	-	-
Injury, n (%)	4 (40.0%)	-	-
Failure, n (%)	-	9 (90.0%)	10 (100.0%)
Loss, n (%)	-	-	-
ESKD, n (%)	-	-	-

SG: Sham Group; CG: Control Group (also submitted to ischemia-reperfusion of the left kidney); CsAG: Cyclosporine A Group (submitted to the same CG procedures plus intraperitoneal CsA at a dose of 15 mg.kg ^1^ ); ESKD: end-stage kidney disease.

**Figure 4:**
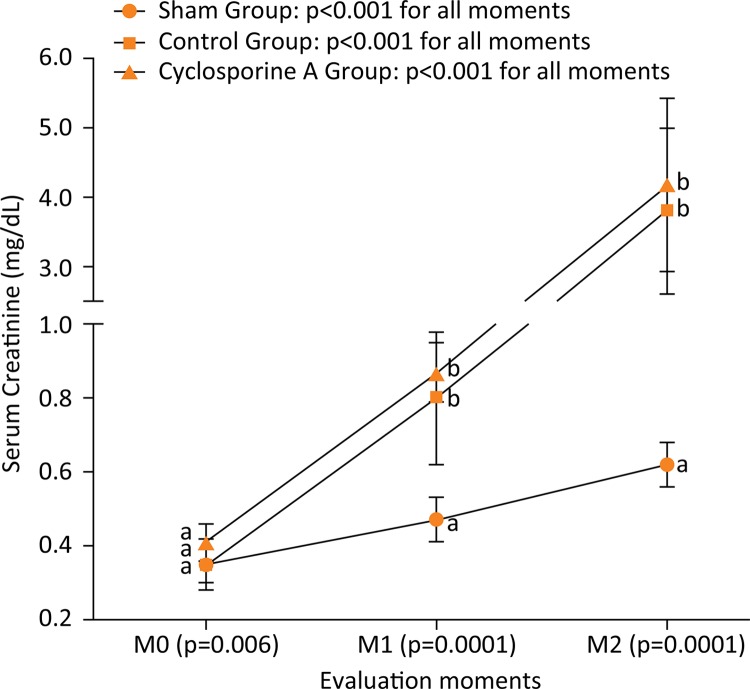
Comparison (intragroup analysis) between the three evaluation moments of serum creatinine from Wistar rats (n=30) submitted to laparotomy and right nephrectomy. Legend: Different letters represent statistical differences between groups (intergroup analysis) of Bonferroni post-hoc test. Control Group is also submitted to ischemia-reperfusion of the left kidney; Cyclosporine A Group is submitted to the same procedures of control group plus intraperitoneal Cyclosporine A at a dose of 15 mg.kg ^1^ ; M0: following catheterization of the left carotid artery; M1: 30 minutes after releasing the left renal artery; M2: 12 hours following the conclusion of the initial surgical procedure.

With respect to renal function, RM ANOVA measurements of serum creatinine (F(2.81)=161.2 and p<0.0001) followed by Bonferroni’s post-hoc indicated that means in the M0 was lower than in M1 and then in M2 in all groups using. In addition, mean values of M1 were also lower than values observed in M2 for all groups ( Fig. 4 ).

**Figure 5:**
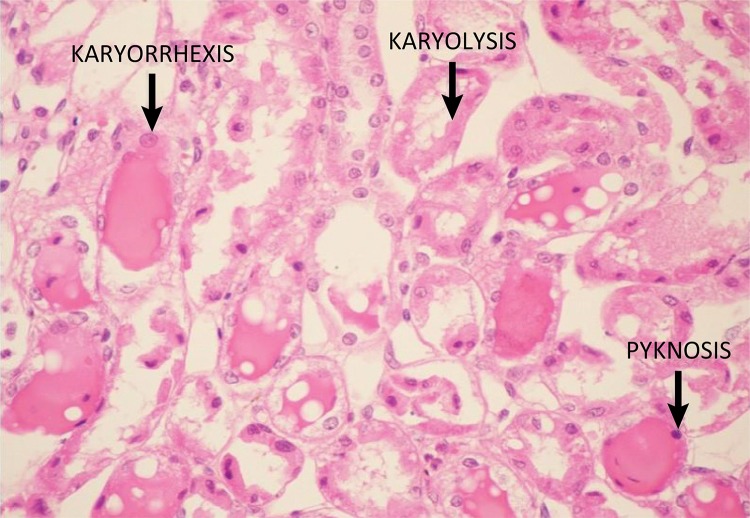
Histological slide demonstrating renal tubular necrosis marks in this present study which supports the Park Evaluation (image is increased by x400).

Histological assessments produced marked results, with approximately half of the CsAG animals classified as moderate to severe (25 to 50% of the histological surface with tubular necrosis), in contrast to the rats in CG, most of which had very severe lesions (>75% of the histological surface showing tubular necrosis) ( Table 7 and Fig. 5 ).

**Table 7:** Histological evaluation in Wistar rats (n=30) submitted to laparotomy and right nephrectomy.

Histology	GROUPS		
	SG (n=10)*	CG (n=10)	CsAG (n=10)
**Right Kidney**			
No Lesion	10 (100.0%)	10 (100.0%)	10 (100.0%)
**Left Kidney**			
No Lesion	8 (88.9%)	-	-
Mild, n (%)	1 (11.1%)	1 (10.0%)	-
Moderate, n (%)	-	-	-
Moderate to Severe, n (%)	-	-	4 (40.0%)
Severe, n (%)	-	-	1 (10.0%)
Very Severe, n (%)		9 (90.0%)	5 (50.0%)

SG: Sham Group; CG: Control Group (also submitted to ischemia-reperfusion of the left kidney); CsAG: Cyclosporine A Group (submitted to the same CG procedures plus intraperitoneal CsA at a dose of 15 mg.kg ^-1^ ). *One left kidney removed was not evaluated by loss during standard storage process in the experimental facility.

## Discussion

The present study aimed to evaluate the effects of CsA on ischemia-reperfusion injury in rat kidneys. Our results indicated that the administration of this drug did not, at the specified dosage and under the conditions described herein, prevent tissue damage arising from ischemic injury following reperfusion. Despite the statistically significant differences between rodents’ weight in the three groups, this parameter did not interfere on the results from other studied variables, as all of them belong to the same lineage and have the same degree of maturity. In addition, the distribution between groups was by random draw.

Our report involved the evaluation of validated measures and scales commonly used in the investigation of kidney injury, including measurements of plasma levels of creatinine ^8 , 16^ and urea ^8^ , as well as RIFLE criteria and histological analysis ^2 , 14 , 16^ . In summary, the results obtained herein were statistically equivalent with respect to each measure considered, with substantial consistency observed among the studied groups. Although the Sham group, under paired analysis, exhibited significant variation in serum creatinine values, non-paired analysis of creatinine measures among the three groups showed significant differences between the Sham and Control groups and the Sham and Cyclosporine groups. These findings were indeed reinforced by descriptive analysis of RIFLE criteria, under which almost all animals in the Cyclosporine and Control groups exhibited renal failure. Histological evaluation was the sole exception, revealing that while nearly half of the CsA animals exhibited tubular necrosis along 25 to 50% of the histological surface examined, almost all Control animals demonstrated 75% or more tubular necrosis.

A very recent study shows a different result, suggesting that CsA protects against renal IRI in a dose-dependent manner, improving renal function and morphology, probably mediated by inhibition of mitochondrial permeability transition pore (mPTP), which would explain why the concentration of CsA has to be high at the time of reperfusion. This study used CsA injected at different times and with different doses, namely 3 mg.kg ^-1^ , 1 hour or 10 minutes before 30 minutes ischemia or 10 mg.kg ^-1^ , 10 minutes before the same ischemia pattern ^16^ . The same study points out the simplicity of pharmacological renal conditioning using different doses and timings of CsA injections whilst contributes importantly to a very sparse literature on this subject ^8 , 11 , 17^ .

As has been previously reported, time and dosage are elements crucial to final outcomes ^13^ . It is worth noting that additional important factors, both known and unknown, can also contribute to the variability observed in the experimental study results, including the heterogeneity of evaluations, time of ischemia, drug dosage, route of administration, time of administration, associated interventions, target organ and the presence of unrelated diseases, among other aggravating factors ^8 , 10 , 11 , 14 , 18 - 21^ . Nevertheless, under descriptive assessment, analysis of the present RIFLE classification results follows a similar pattern, thusly reinforcing our conclusion that, under the present experimental study conditions, the administration of cyclosporine was insufficient to prevent ischemia-reperfusion injury in rat kidneys, despite seeming to offer some degree of protective benefit as evidenced by the histological evaluation.

Inferential analyses of creatinine indicated a worsened pattern of results in both Control and Cyclosporine groups in comparison to the Sham group, similar to what has already been partially described in the literature ^13^ . In contrast, a well-designed study demonstrated different results in the heart ^21^ , yet the intervention in this case consisted of cyclosporine and post-conditioning. Interestingly, few studies have attempted to conduct dose-response curve analyses. A previous study performed in the USA evaluating differences between the use of CsA and a 20% lipid solution showed that, according to the dose-response curve, the latter proved more effective at preventing cardiac injury ^6^ in accordance with infarct area size. Taken together, it may be worth suggesting future studies endeavoring to evaluate CsA dose-response curves to be performed with the objective of elucidating whether cyclosporine is indeed unable to prevent renal injury, or whether methodological variability and study design are responsible for the controversial evidence presented with respect to different organs ^11 , 16 , 17^ .

The present study suffered from the following limitations: the exclusion of additional assessment measures, including other biomarkers, follow-up to investigate long-term effects and the lack of a CsA control group. Although the dose of CsA used is usually considered nephrotoxic, the fact that CsA was administered as a double single bolus 24 hours apart reduces the risk of vascular toxicity. Indeed, although CsA increases renal vascular resistance and decreases renal blood flow, the hemodynamic impact is transient ( *<* 10 min) as has been demonstrated experimentally by other groups ^22 , 23^ .

It is important to emphasize even though this study revealed an inability of CsA to prevent the deleterious effects of ischemia-reperfusion injury in rodent kidney in, it is possible to hypothesize some potential beneficial action of the CsA in this setting as the present histological analysis results suggest a protective effect. It is worth noting that the failure to prove total prevention of kidney injury by ischemia and reperfusion in this case poses no impediment to further study. It is our hope that the results presented herein, taken together with those presented by other reports in the literature, may aid in better defining the conditions under which these types of interventions should be realized.

## Conclusion

CsA was unable to prevent the deleterious effects of ischemia-reperfusion injury in rat kidneys under the conditions of the present study.
